# Temporal and spatial trends of fentanyl co-occurrence in the illicit drug supply in the United States: a serial cross-sectional analysis

**DOI:** 10.1016/j.lana.2024.100898

**Published:** 2024-09-27

**Authors:** Tse Yang Lim, Huiru Dong, Erin Stringfellow, Zeynep Hasgul, Ju Park, Lukas Glos, Reza Kazemi, Mohammad S. Jalali

**Affiliations:** a Center for Communicable Disease Dynamics, Harvard T.H. Chan School of Public Health, Boston, MA, USA; b Massachusetts General Hospital Institute for Technology Assessment, Harvard Medical School, Boston, MA, USA; c Warren Alpert Medical School, Brown University, Providence, RI, USA; d Center for Drug Evaluation and Research, U.S. Food and Drug Administration, Silver Spring, MD, USA

**Keywords:** Illicit drug supply, Fentanyl co-occurrence, Substance use, Temporal and spatial trends

## Abstract

**Background:**

Fentanyl and its analogs contribute substantially to drug overdose deaths in the United States. There is concern that people using drugs are being unknowingly exposed to fentanyl, increasing their risk of overdose death. This study examines temporal trends and spatial variations in the co-occurrence of fentanyl with other seized drugs.

**Methods:**

We identified fentanyl co-occurrence (the proportion of samples of non-fentanyl substances that also contain fentanyl) among 9 substances or substance classes of interest: methamphetamine, cannabis, cocaine, heroin, club drugs, hallucinogens, and prescription opioids, stimulants, and benzodiazepines. We used serial cross-sectional data on drug reports across 50 states and the District of Columbia from the National Forensic Laboratory Information System, the largest available database on the U.S. illicit drug supply, from January 2013 to December 2023.

**Findings:**

We analyzed data from 11,940,207 samples. Fentanyl co-occurrence with all examined substances increased monotonically over time (Mann-Kendall *p* < 0.0001). Nationally, fentanyl co-occurrence was highest among heroin samples (approx. 50%), but relatively low among methamphetamine (≤1%), cocaine (≤4%), and other drug samples. However, co-occurrence rates have grown to over 10% for cocaine and methamphetamine in several Northeast states in 2017–2023.

**Interpretation:**

Fentanyl co-occurs most commonly with heroin, but its presence in stimulant supplies is increasing in some areas, where it may pose a disproportionately high risk of overdose.

**Funding:**

This work was partly supported by 10.13039/100000038FDA grant U01FD00745501. This article reflects the views of the authors and does not represent the views or policies of the FDA or US Department of Health and Human Services.

**Research in context**

**Evidence before this study**

Fentanyl and its analogues are the major driver of rapidly increasing overdose mortality. Overdose mortality data increasingly show the presence of multiple drugs with fentanyl, including stimulants such as methamphetamine and cocaine, raising concern about unintentional fentanyl exposure among users of other drugs. We searched PubMed titles and abstracts for original research articles examining the U.S. illicit drug supply for evidence of fentanyl contamination or adulteration of methamphetamine, cocaine, and other drugs. We used the following search terms (contaminat∗ OR adultera∗ OR combin∗ OR “drug check∗”) AND fentanyl AND (methamphetamine OR cocaine OR ecstasy OR MDMA OR “club drugs” OR cannabis OR marijuana OR benzo∗ OR amphetamine OR “LSD” OR hallucinogen∗) AND (sample OR supply). We excluded commentaries, articles reporting user perception of the drug supply, and studies using human sample toxicology testing. We did not restrict by date. Only one study examined national data, for the years 2011–2016, before fentanyl had become as widespread as it is now. Two studies of drug-checking programs were limited to single states, while an additional study reported data across 25 states. There is also publicly available data from drug-checking programs. Collectively, these studies and data report that 0–13% of expected methamphetamine samples, 6.6–25% of powder cocaine samples, and 0–2% of crack/crystalline cocaine samples were positive for fentanyl by more than trace amounts. However, these data and drug-checking studies rely on data obtained during or after 2020 and do not examine trends over time. No national analysis has been conducted to examine the frequency of fentanyl co-occurrence with multiple classes of seized drugs, nor how that has changed over time and across all 50 states.

**Added value of this study**

This study uses the largest and most comprehensive national dataset available on the U.S. illicit drug supply, the National Forensic Laboratory Information System (NFLIS). Analyzing data from January 2013 to December 2023, we examine how fentanyl co-occurrence with other seized drugs varies over space (across all 50 states) and time. We find that fentanyl co-occurrence is common in heroin samples (40–50% by 2022–23) but is generally rare for other substances (under 4% for cocaine, methamphetamine, and cannabis), though it has increased significantly. Co-occurrence varies substantially by region, reaching over 10% for cocaine and 20% for methamphetamine in some northeastern states in some years.

**Implications of all the available evidence**

All available evidence suggests that unintentional exposure to fentanyl is a real risk, but one that differs by drugs used and region. The present study draws on NFLIS data, which is limited by its inclusion of seized drugs, the composition of which could change over time due to shifts in law enforcement priorities and policy. Nonetheless, in conjunction with other sources, such as local drug-checking programs, this study highlights which drugs are most likely to put people at risk of accidental fentanyl exposure. These findings could support more targeted harm reduction efforts.

## Introduction

Over 106,000 people in the United States died of drug overdoses in 2021.^1^ Deaths have more than doubled since 2013 (44,000), when illicitly manufactured fentanyl entered the drug supply,^2^ driving rapid growth in overdose mortality.^1^ While illicit fentanyl has primarily been associated with the heroin supply,^2^ there is growing concern about its presence in other illicit drug supplies, such as cocaine, methamphetamine, prescription opioids, and benzodiazepines.^3^ Indeed, overdose deaths co-involving fentanyl and other substances besides heroin have increased nearly 60-fold from 2010 to 2021 nationally.^4^

Fentanyl is highly potent (and some fentanyl analogs even more so),^5^ so unintentional exposure to fentanyl via contamination of other substances could considerably elevate the risk of overdose and death, potentially contributing to the growth in mortality. Such contamination could occur at many places in the illicit drug supply chain, such as through accidental or deliberate mixing in production facilities or careless handling by dealers.

There are few comprehensive, up-to-date analyses focusing on recent trends in fentanyl co-occurrence in the illicit drug supply, especially by substance and geography. Research and publicly available data from drug-checking programs (where users submit samples for testing) suggest that fentanyl *is* sometimes unexpectedly present in other drug supplies: these studies and data, mostly from 2020 onwards, report between 0% and 38% of expected methamphetamine samples, between 2.1% and 23% of powder cocaine samples, and between 0% and 2% of crack/crystalline cocaine samples were positive for fentanyl by more than trace amounts.^6^, ^7^, ^8^, ^9^, ^10^ However, these studies use data from limited states; furthermore, as robust drug-checking programs have only existed since 2020–2021,^7^ they are unable to examine temporal trends in fentanyl's spread.

To understand the threat of fentanyl, it is critical to quantify temporal and spatial trends in its co-occurrence with other substances in the illicit drug supply. While such co-occurrence is several steps removed from the key outcome of overdose mortality, it provides an approximate floor value or minimum for the risk of unintentional fentanyl exposure. The only national analysis examining the frequency with which fentanyl co-occurs with multiple classes of seized drugs used data from 2011–2016.^11^ While this study did find that fentanyl co-occurrence with stimulants increased significantly during this time, these seizures reflect the illicit drug supply just as fentanyl was beginning to supplant heroin. More recent data show increasing fentanyl-containing drug seizures between 2018 and 2021 in High Intensity Drug Trafficking Areas (areas designated as being important for illicit drug supply, which receive enhanced law enforcement resources and coordination), though without examining fentanyl co-occurrence with other substances.^12^ There has not been a recent analysis of the co-occurrence of fentanyl in other seized drugs at the national level and over time.

This study aims to address this critical gap using national data, spanning 2013–2023, to quantify temporal trends and state-level spatial variation in the co-occurrence of fentanyl and fentanyl-related substances (hereafter, fentanyl) with other seized drugs.

## Methods

### Data

We used data from the National Forensic Laboratory Information System (NFLIS-Drug), which aggregates drug chemistry analysis results voluntarily reported from forensic laboratories analyzing law enforcement drug seizures across the U.S. This study analyzes drug reports submitted to laboratories from January 2013 to December 2023, that were analyzed and reported by July 26, 2024. All NFLIS-Drug data were obtained from the U.S. Drug Enforcement Administration (DEA) utilizing a memorandum of understanding between the U.S. Food and Drug Administration (FDA) and the DEA.

Record-level NFLIS-Drug data consist of drug reports, each representing a single instance of a specific substance detected,^13^ including state, county, month, and year of seizure, and unique seizure-level and item/sample-level identifiers. Generally, each *seizure* represents a single incident of law enforcement confiscating suspected drugs; a seizure can potentially result in multiple *items/samples* being submitted for forensic analysis, each of which in turn contains one or more substances, with each substance detected constituting a single drug *report* (Supplementary Figure S1).^14^ The seizure- and item/sample-level identifiers allow us to differentiate between scenarios where different substances co-occur in the same seizure but different samples (such as separate packages found in the same seizure) and cases where they co-occur in the same sample (such as multiple substances packaged together), which our analysis focuses on. Up to eight substances are reported for each item/sample.^14^

NFLIS-Drug data have several important limitations, discussed further in *Limitations* below and in Supplementary Methods. It nevertheless remains the largest, most comprehensive source of data on the national illicit drug supply, which is otherwise not systematically monitored.

### Analysis

Our primary outcome of interest is the co-occurrence of fentanyl and fentanyl-related substances with other illicit substances, which we define as the percentage of samples of any given non-fentanyl substance that also contain fentanyl, i.e., that share an item/sample-level identifier with a fentanyl record. Note that this differs from the co-occurrence of other substances with fentanyl, where the denominator would be the number of samples of fentanyl rather than of the other substances.

We focus on fentanyl co-occurrence with illicit substances that people who use drugs would typically seek out for consumption, such as heroin or methamphetamine. Street drugs often contain multiple active substances, and consumers are rarely aware of everything that is in their product. Notwithstanding that the true composition of the illicit drug supply is rapidly evolving, generally, consumers still refer to and seek drugs by informal categories such as ‘dope’, ‘crack’, or ‘weed’, or occasionally more specific prescription products.^6^^,^^7^^,^^10^ Note that limiting our focus to fentanyl co-occurrence with desired substances in this way excludes cases like counterfeit prescription medications consisting of different active ingredients from their genuine counterparts (e.g., bromazolam in counterfeit Xanax), as well as novel psychoactive substances increasingly co-occurring with fentanyl (e.g., isotonitazene), which are beyond the scope of this analysis.

To identify non-fentanyl substances of interest, we extracted the 60 most frequently reported drugs for each year from 2013 to 2023 from NFLIS annual reports into a combined list. From this list, we identified nine substances or substance categories (hereafter ‘substances examined’ or simply ‘substances’) typically sought after by people who purchase or consume drugs: methamphetamine, cannabis and other cannabinoids (hereafter ‘cannabinoids’), cocaine, heroin, prescription opioids, cocaine, methamphetamine, prescription benzodiazepines, prescription stimulants, hallucinogens, and club drugs (e.g., ketamine, MDMA). We excluded non-psychoactive substances, such as testosterone, ibuprofen, levamisole, and naproxen. Substance categories accounted for chemical similarities (e.g., salts, isomers, analogs) and alternative names. Some substance categories are labelled as ‘prescription’ based on legal availability of substances included; actual samples analyzed and reported may be illicitly manufactured, as NFLIS-Drug does not identify sources of substances. Supplementary Table S1 presents the complete list of substances in each category and Supplementary Table S2 shows category frequencies. Separately, we identified reports of fentanyl and fentanyl-related substances, including reports of fentanyl only as well as fentanyl seized with other substances (see Supplementary Table S2).

At the national level, we calculated the monthly percentage and 12-month moving average of fentanyl co-occurrence with each of the other substances examined. To identify geographic variation, we further examined yearly trends in fentanyl co-occurrence with these substances, aggregated and separately, at the state level. We assessed the direction and significance of trends in observed fentanyl co-occurrence rates using the Mann-Kendall trend test, a non-parametric method designed to detect monotonic increases or decreases over time.

Analyses were conducted in R, version 4.3.1. The analysis code is available online at https://github.com/tseyanglim/NFLIS-analysis. The Mass General Brigham institutional review board exempted the study from review and waived informed consent. We followed STROBE reporting guidelines (see Supplementary Table S3).

### Role of the funding source

The funding organization (FDA) had no role in the design and conduct of the study; analysis and interpretation of the data; preparation of the manuscript; and decision to submit the manuscript for publication. FDA assisted in obtaining data from DEA, utilizing a memorandum of understanding between FDA and DEA, and also reviewed the manuscript before submission.

## Results

The dataset comprises 17,759,878 drug reports from 2013 to 2023, that were analyzed and reported by July 26, 2024, across 50 states and the District of Columbia. These reports comprised 16,658,920 samples from 9,584,522 seizures. Frequencies of seizures, samples, and drug reports in the full dataset are in Supplementary Figure S1.

The nine substances examined were found in 11,940,207 samples, and fentanyl co-occurred in 214,899 (1.8%) of these samples. About 95% of the samples contained only one substance (Supplementary Figure S1). Among the 672,604 samples containing multiple drug reports, an average of 2.3 (standard deviation: 0.7) substances were detected per sample. The total number of samples and samples with co-occurring fentanyl for each substance examined are presented in Supplementary Table S2.

**Fig. 1:**
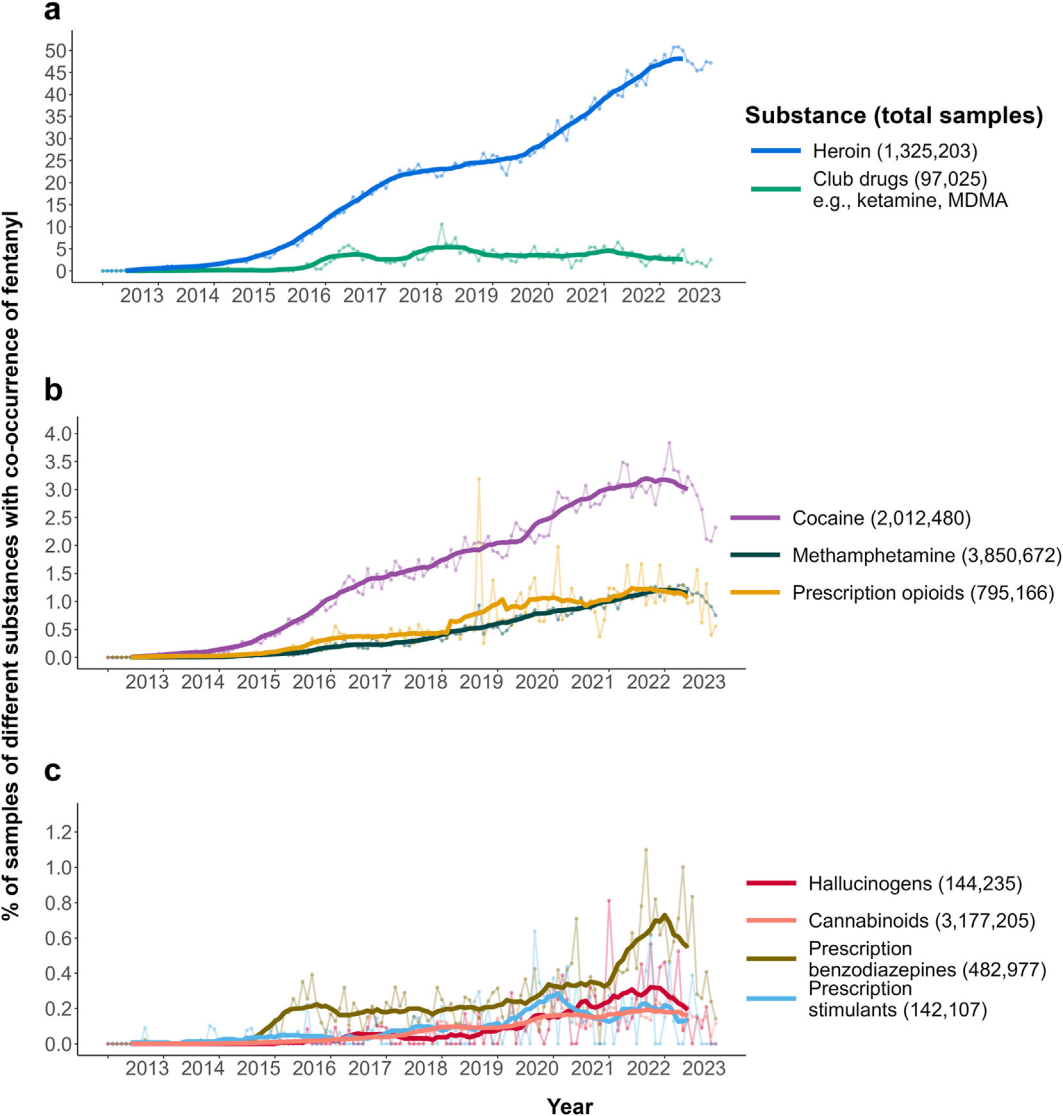
*National trends in the proportionof different substance samples with co-occurring fentanyl in the United States, 2013–2023, showing monthly (dots and faint lines) and 12-month centered moving average monthly proportions (thick lines). Note the differing y-axis scales*.

Fig. 1 shows national-level trends in the monthly proportion of samples with co-occurring fentanyl. Overall, fentanyl co-occurrence increased significantly over time across all substance classes (Mann-Kendall *p* < 0.0001 for all 9 substances; Supplementary Table S4). Samples with reported heroin have consistently had the highest proportion of co-occurring fentanyl, increasing from 0% in early 2013 to 20% by 2018 and about 50% in 2023 (Fig. 1A). Fentanyl co-occurrence with club drugs (e.g., MDMA) rose from approx. 0%–5% from 2016 to 2019 and stayed at that level since, though with some variation over time. Fentanyl co-occurrence with methamphetamine and cocaine (Fig. 1B) has been increasing but remains under 4%. By 2023, fentanyl was detected in less than 4% of cocaine samples and around 1% of methamphetamine samples, up from <0.05% to <0.01% respectively in 2013. Fentanyl co-occurrence with prescription opioids reached over 1% by 2023, though also with substantial variation over time (Fig. 1B). Fentanyl co-occurrence with cannabinoids remained below 0.3% over the study period (Fig. 1C), while co-occurrence with hallucinogens, prescription stimulants, and prescription benzodiazepines varied substantially but was largely ≤1% (Fig. 1C).

**Fig. 2:**
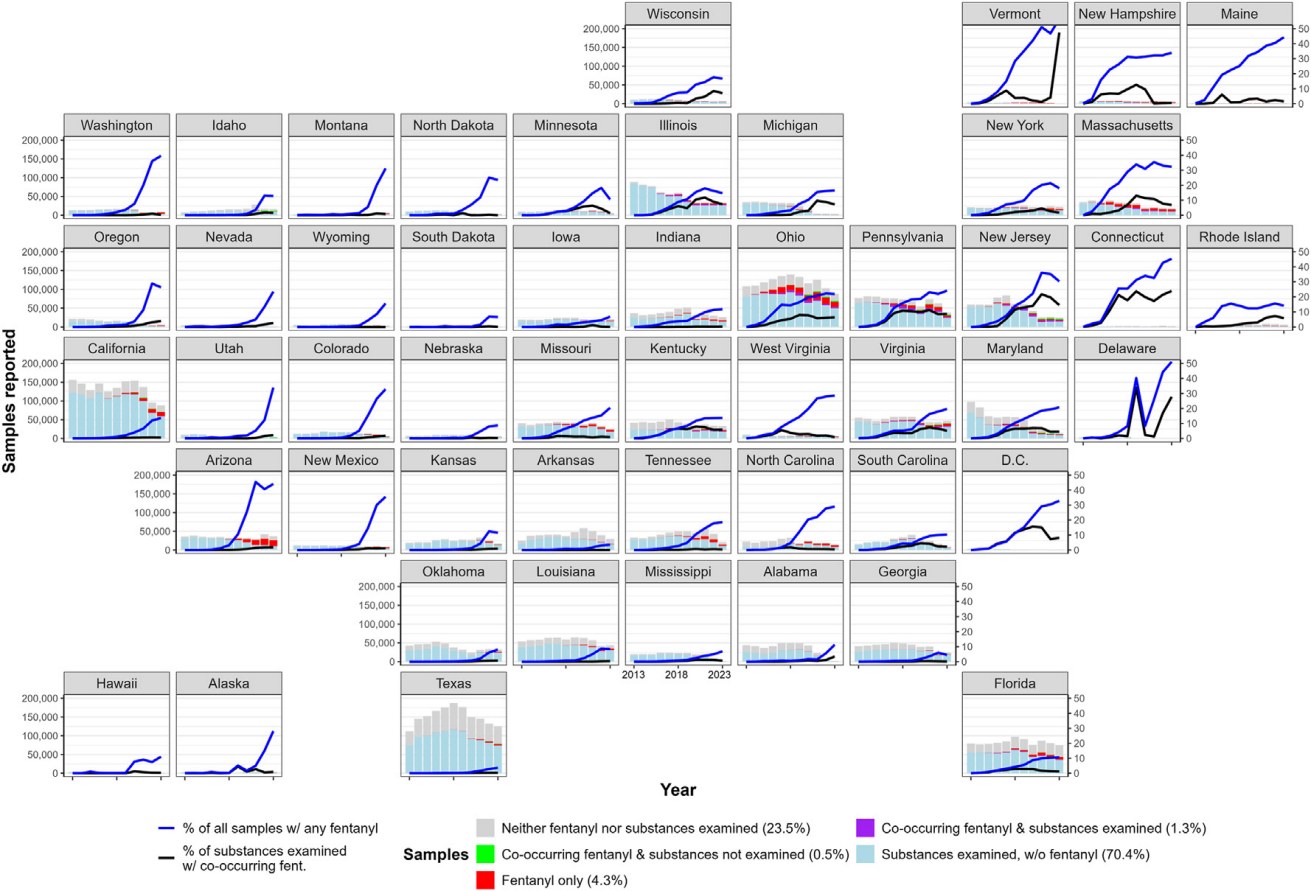
*State-level annual totals of samples analyzed and reported, and trends in the proportion of fentanyl co-occurrence in the United States, 2013–2023. Data points for % of fentanyl among total samples in VT (year 2021: 51.1%; year 2023: 56.6%) and DE (year 2023: 51.1%) were excluded from the figure*.

Fig. 2 shows state-level annual totals of samples analyzed and reported, contrasting numbers of samples without vs. with co-occurring fentanyl. It also highlights that most fentanyl (70.4% of fentanyl-containing samples) is reported on its own, without any other substances. For context, the map also shows the total number of samples of substances we did not examine (grey bars, 23.5% of all samples). Fig. 2 also shows two trends: the percentage of all samples containing any fentanyl, and the percentage of samples of substances examined with co-occurring fentanyl.

There is large geographic variation. California, Texas, and Ohio consistently report the most samples analyzed, while a handful of other states (e.g., Florida, Pennsylvania, Illinois, New Jersey, Louisiana) also report substantial numbers of samples. The percentage of samples reported as containing fentanyl has increased in nearly all states, starting around 2013 in the Northeast (e.g., Massachusetts, Maine) and Midwest (e.g., Ohio, Illinois, Minnesota) and starting later (2017–2020) elsewhere; as of 2021, fentanyl is reported in over 10% of samples in most states outside the Gulf Coast (e.g., Texas, Mississippi). While the frequency of samples with only fentanyl reported has increased across the board, the frequency of samples with co-occurring fentanyl has not increased uniformly. Such co-occurrence has increased in the Northeast and Midwest, with slight upticks since 2021 in a few western states (e.g., Oregon, Nevada), but remains under 5% across the Gulf Coast and most western states despite the increasing frequency of fentanyl samples reported. In states like Washington, Arizona, or New Mexico, for instance, fentanyl comprised nearly 40–50% of all samples in 2023, but co-occurred in <5% of samples of substances examined.

**Fig. 3:**
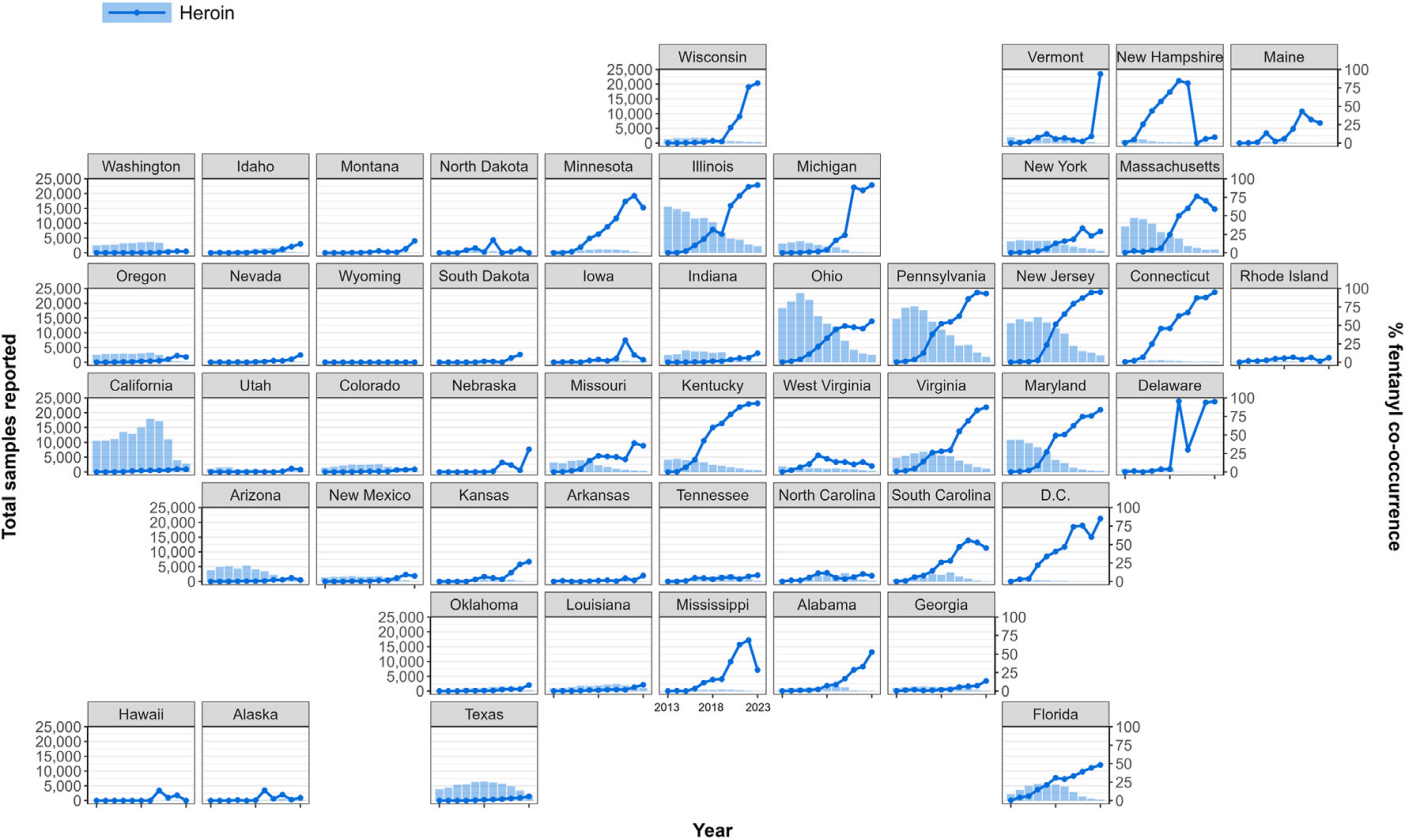
*State-level trends in the yearly proportion of heroin samples with co-occurring fentanyl in the United States, 2013–2023. The following data points were excluded from the figure as the number of total tested samples was ≤5, which caused unstable estimates. For heroin: DE (year: 2021), ME (year: 2023), and SD (2023)*.

Fig. 3 shows state-level trends in fentanyl co-occurrence with specific substances: heroin, cocaine, and methamphetamine. Fentanyl co-occurrence with heroin was highest in the east of the country, particularly in the Northeast (e.g., New Jersey, 95.1% in 2023) and Midwest (e.g., Michigan, 91.4% in 2023), but virtually zero across the West Coast even in recent years (Fig. 3).

**Fig. 4:**
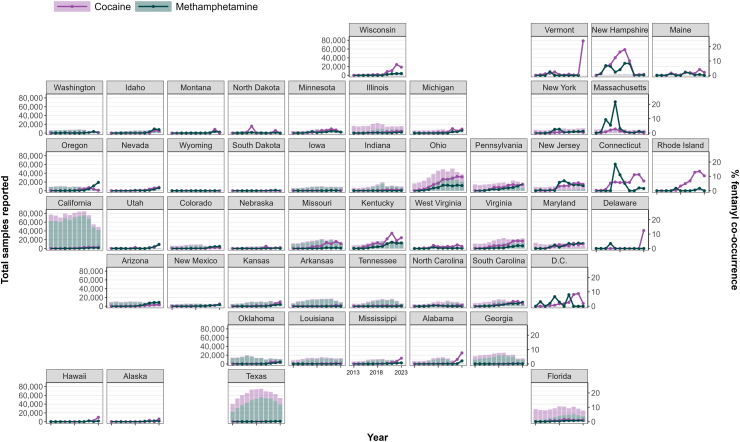
*State-level trends in the yearly proportion of cocaine and methamphetamine samples with co-occurring fentanyl in the United States, 2013–2023. Data for cocaine for VT (2023) was excluded from the figure as the number of total tested samples was ≤5, which caused unstable estimates*.

While fentanyl co-occurrence with cocaine was ≤4% nationally, parts of the Northeast had higher rates, with recent co-occurrence rates over 10% in New Hampshire, Connecticut, and Rhode Island. Kentucky and Ohio also had similarly above-average levels of co-occurrence with cocaine (Fig. 4). Fentanyl co-occurrence with methamphetamine showed similar geographic concentration, arising mostly in Massachusetts (21.7% in 2017), New Hampshire (over 8.0% from 2019 to 2020), Connecticut (over 10.0% from 2017 to 2018), and New Jersey (7.0% in 2019). State-level trends (Supplementary Table S5) in fentanyl co-occurrence with club drugs, cannabinoids, prescription opioids, hallucinogens, prescription stimulants, and prescription benzodiazepines generally remained under 2%, albeit with occasional spikes in some states and years (Supplementary Figure S2), particularly for club drugs and, more recently, cannabinoids in some northeastern and Appalachian states (e.g., Kentucky).

## Discussion

Fentanyl co-occurrence has increased significantly across all nine substances examined from 2013 to 2023, though co-occurrence rates and magnitudes of increase vary widely by substance and region.

Unsurprisingly, fentanyl co-occurrence was most common in heroin, accounting for the majority of samples (Supplementary Table S4) with co-occurring fentanyl. Heroin contamination with fentanyl is well-documented, especially in the east where powder-form fentanyl is easily mixed with white powder heroin.^2^^,^^15^ Our results corroborate these trends; in the western areas, which are dominated by black tar heroin, fentanyl co-occurrence with heroin in NFLIS-Drug samples was low (<5%) through 2023, even though reports of fentanyl itself have been increasing. These West Coast reports likely reflect fentanyl on its own, often in pill form,^12^ which presents its own risk independent of contamination of other drugs, especially to unsuspecting prescription pill users. Fentanyl co-occurrence with heroin drives most of the geographic pattern observed in fentanyl co-occurrence more broadly.

Fentanyl co-occurrence with other non-opioid substances, while also increasing, remained low overall during the study period. Nationally, as recently as 2023, under 5% of cocaine (2.7%), methamphetamine (1.0%), prescription medications ( ≤ 1.0%), or cannabinoid samples (0.4%) had co-occurring fentanyl. National aggregate co-occurrence with these substances also appears to be plateauing or starting to decline in late 2023, though it is too soon to distinguish a genuine trend shift from random fluctuation. While NFLIS-Drug data may understate the extent of fentanyl co-occurrence with other substances downstream in the illicit supply chain, our findings suggest that fentanyl contamination of the illicit non-opioid supply may be less prevalent nationally than is perceived.^3^ However, these national averages mask regional trends (Supplementary Table S2). In parts of the Northeast, Kentucky, and Ohio, fentanyl co-occurred with over 10% of cocaine samples by 2021 (vs. ∼3% nationally); these tend to be areas where fentanyl and cocaine are relatively common in the first place, on their own or mixed with heroin. Some northeastern states also had high rates of fentanyl co-occurrence with methamphetamine—over 10% of methamphetamine samples in some states and years (vs. ∼1% nationally)—albeit with substantial variability over time. Unlike cocaine, methamphetamine is generally uncommon in the Northeast (though increasingly present in recent years).^16^ With relatively widespread fentanyl and rare methamphetamine, the proportion of fentanyl co-occurrence in these regions was sensitive to the small denominator. These findings are generally consistent with drug-checking data, which draws disproportionately from Northeastern states. For instance, StreetCheck reported data from Massachusetts in 2021, finding fentanyl to be present in 14.15% of samples in which the primary illicit substance was cocaine and 14.29% of samples in which the primary illicit substance was methamphetamine.^7^

Low rates of fentanyl co-occurrence with stimulants in seized drugs contrast with, but do not contradict, growing mortality rates co-involving fentanyl and stimulants.^4^ Drugs consumed together (and hence potentially co-involved in overdose) are not necessarily ever physically mixed. Supply data are thus only one piece of the puzzle of growing polysubstance mortality.

Notwithstanding its rarity, fentanyl co-occurrence with non-opioids is likely disproportionately dangerous. Many people exposed to fentanyl this way may be opioid-naïve, or at least not taking appropriate mitigation measures for potential opioid overdose, and hence at higher risk of death.^17^ Furthermore, the unpredictability of fentanyl occurrence is itself an additional risk factor, making behavioral adaptations to manage risk more difficult.^2^ As such, even these low rates of fentanyl co-occurrence cause concern. Even a little contamination could be sufficient to increase mortality substantially given stimulant users’ low tolerance. The general increase of fentanyl co-occurrence across substance classes may reflect an increasing risk of accidental or deliberate contamination as fentanyl proliferates.

Additionally, fentanyl is increasingly co-occurring with emerging new psychoactive substances such as xylazine, with increasing co-involvement in overdose deaths.^18^ Here we focused on fentanyl co-occurrence with substances salient to people who purchase or consume drugs; future research could focus on exploring the important emerging threat of novel psychoactive substances mixed with fentanyl. Future work could also examine the drivers of increasing fentanyl co-occurrence, such as shifts in trafficking routes, market dynamics, and consumer preferences, to inform strategies to address that increase.

Our work underscores the need for multifaceted approaches to mitigate the impacts of fentanyl's ongoing spread. Strengthened monitoring of the illicit drug supply is paramount, offering granular, timely awareness of fentanyl exposure risk and allowing appropriate precautions such as naloxone distribution to people who may engage in or witness drug use.^19^ Increased access to evidence-based treatment such as methadone and buprenorphine will also greatly reduce the risk of overdose among patients with opioid use disorder.^20^ Broader distribution of fentanyl test strips, including to people who use benzodiazepines and stimulants rather than just people who intentionally use fentanyl, would help^21^; better still, community drug checking programs using higher-precision analytical equipment to track relative concentrations in drugs actually being used (not seized) could provide a localized early warning system.^21^^,^^22^ Such measures would also help with addressing the ongoing opioid crisis—fentanyl substitution of heroin is the major driver of uncertainty in projections of opioid overdose mortality over the next decade,^23^ and mitigating fentanyl's impact on overdose risk is a high-leverage intervention point.^24^

A more comprehensive and sustainable approach would be systematic, national-level, real-time drug monitoring efforts by public health agencies. Systematic monitoring would allow standardization of analyses, reporting, and communication. For example, the Rapid Analysis of Drugs program in Maryland and the Massachusetts Drug Supply Data Stream have supported public health responses to overdose deaths by identifying and rapidly communicating changes in the illicit drug market.^25^^,^^26^ It would also be useful to separate the public health function of monitoring from the law enforcement function of the existing NFLIS-Drug system, as the two have different goals. Public health drug monitoring aims above all to provide an accurate understanding of the composition of the illicit drug supply to facilitate behavioral adaptation, whereas law enforcement efforts are aimed at disrupting and reducing supply rather than informing consumers. People who use drugs are also less likely to engage with or trust law enforcement services for fear of arrest or prosecution, impeding monitoring efforts. Improving public health monitoring could have important benefits, but will be challenging given the complex legal and social environment in the United States.^27^

### Limitations

Our analysis has limitations. First, without reliable data on substance quantity, purity, and region-specific qualitative details (see Supplementary Methods), it is difficult to infer the nature of and reasons for fentanyl co-occurrence,^17^ such as the risk it poses, where in the illicit supply chain mixing occurs, and whether mixing appears to be accidental or intentional.

Second, NFLIS-Drug data lack information on original drug identities (e.g., ‘seized as’ or ‘sold as’), which could further elucidate whether co-occurrence arises intentionally or accidentally, whether consumers are aware of potential fentanyl presence, and which populations of people who use drugs may be at risk. It could also help explain differences between perceptions and reality of the risk of fentanyl exposure.^28^ Furthermore, fentanyl (without co-occurring substances) being presented or sold as something else creates a risk of unintentional exposure not captured by our co-occurrence metrics.

Third, our analysis aggregates fentanyl and fentanyl-related substances, including precursors, analogues, and derivatives (see Supplementary Table S1). Some fentanyl analogues are far more potent than even fentanyl (e.g., carfentanil), and their co-occurrence with other substances would be particularly concerning. Distinguishing co-occurrence rates of specific analogues or related substances would be a useful future research direction.

Finally, seizures data have inherent limitations. Despite NFLIS-Drug being the most comprehensive database available to researchers, it may not fully reflect the broader illicit drug supply due to potential non-representativeness and irregular data reporting.^14^ Seizures may disproportionately occur upstream in the supply chain, and thus may not reflect the composition of drugs at the level of actual consumption, with additional mixing likely occurring further downstream. Furthermore, drugs seized are removed from community use.

Variations in laboratory practices, definitions of samples/items, and testing thoroughness can also lead to incomplete or underreported substance identification (see Supplementary Methods). Not all substances seized are analyzed, not all substances present will be detected, and not all substances detected are necessarily reported. These issues are especially applicable for novel or emerging substances (potentially including some less common fentanyl analogues, though not base fentanyl or its more common analogues^29^). Reasons for taking multiple samples from seizures may vary, and the composition of samples within a seizure may not be independent, potentially biasing sample-level counts. Analysis of seizures and reporting of substances may be further biased by different and changing law enforcement priorities and legal standards across time and geography, so regional differences should be interpreted with caution. Absent relevant metadata, we cannot correct or adjust for these changes and variations in our analysis.

Despite these limitations, NFLIS still represents the best available data on the nation's drug supply. Even drug-checking services do not reliably report ‘sold as’ or expected vs. actual substances present, and they are still limited in scope, reporting hundreds to thousands of samples over <5 years, vs. millions of samples over at least 10 years in NFLIS. That our results are relatively consistent with drug-checking data in the regions of the country where fentanyl is most prevalent increases our confidence that NFLIS reflects, at least in part, the composition of drugs as obtained by consumers.

### Conclusions

Findings from this study indicate a consistent and significant increase in fentanyl co-occurrence with various substances across the U.S. from 2013 to 2023. While fentanyl primarily co-occurs with heroin, its presence in stimulant supplies is also rising. Our results show no evidence of widespread fentanyl co-occurrence with cannabis. Regional differences emphasize the necessity of geographically-specific interventions. These findings highlight the critical need for systematic public health drug monitoring to address the growing issue of a contaminated illicit drug supply.

## Contributors

Conceptualization: TYL, JP; Methodology: TYL; HD, EJS, JP; Software: TYL, HD, ZH; Validation: TYL, HD; Formal analysis: TYL, HD; Investigation: TYL, HD, EJS, ZH; Resources: LG, RK, MSJ; Data curation: TYL, HD, ZH, LG; Writing—Original: TYL; Writing—Review & editing: All authors; Visualization: TYL, HD; Supervision, Administration, Funding: RK, MSJ. All authors read and approved the final manuscript. TYL, HD, ZH, LG and MSJ had full access to all data in the study. TYL and MSJ had final responsibility for the decision to submit for publication.

## Data sharing statement

Full analysis code and detailed results tables are available online at https://github.com/tseyanglim/NFLIS-analysis. Record-level NFLIS data used in this analysis may be obtained by request to the DEA.

## Declaration of interests

TYL previously received an ORISE Fellowship from FDA, and has received consulting fees from the Massachusetts General Hospital Institute for Technology Assessment. JP has received consulting fees from FDA, and is a member of the Editorial Board of the International Journal of Drug Policy. LG and RK are employees of FDA. This article reflects the views of the authors and should not be construed to represent the views or policies of the FDA or the US Department of Health and Human Services. The authors declare no further conflicts of interest.
