# Supplementary material for: Temporal and spatial trends of fentanyl co-occurrence in the illicit drug supply in the United States: a serial cross-sectional analysis
Source: Lancet Reg Health Am. 2024 Sep 27;39:100898. doi: 10.1016/j.lana.2024.100898 (PMC11470258; doi:10.1016/j.lana.2024.100898)
Supplement: Supplementary Figures and Tables [file mmc1.pdf]

Supplementary materials for:

Temporal and spatial trends of fentanyl co-occurrence in the illicit drug supply in the United States: A serial cross-sectional analysis

Tse Yang Lim, PhD; Huiru Dong, PhD; Erin Stringfellow, PhD, MSW; Zeynep Hasgul, MSc; Ju Park, PhD; Lukas Glos, MA; Reza Kazemi, PhD; Mohammad S. Jalali, PhD

Online repository at: https://github.com/tseyanglim/NFLIS-analysis

Table of Contents

Supplementary Methods

Supplementary Figure 1: Sample restrictions

Supplementary Table 1: Substance categorizations

Supplementary Table 2: Total reports & samples by substance

Supplementary Table 3: STROBE checklist

Supplementary Table 4: Mann-Kendall test results, monthly

Supplementary Table 5: Mann-Kendall test results, annual by state

Supplementary Figure 2: Additional state-level fentanyl co-occurrence rates

References

Supplementary Methods

NFLIS Data

Many details of the NFLIS program are publicly available online. NFLIS-Drug is the primary component of the program; a detailed discussion of its limitations and potential data issues can be found in [1].

NFLIS-Drug coverage

The NFLIS-Drug database aggregates drug chemistry analysis results voluntarily reported from forensic laboratories analyzing law enforcement drug seizures across the United States. The NFLIS-Drug network includes federal and all 50 state laboratory systems as well as over a hundred local or municipal laboratory systems, nearly 300 individual laboratories altogether.[2] While the NFLIS-Drug network has expanded over time, with a growing number of local or municipal laboratories, the vast majority of laboratories were already participating as of 2013 (the first year of data used in this analysis): 272 in 2013, growing to 284 in 2022.[3,4] The participating laboratories also handle the vast majority of drug analyses conducted nationally: 98.5% in 2022, up from 91% in 2013.[3,4] The reports in the NFLIS-Drug database thus represent a near-complete census of drug chemistry analyses conducted for law enforcement purposes in the United States.

Note that NFLIS-Drug annual summary reports and other aggregated data products typically present statistically adjusted or weighted numbers intended to correct for variation in which laboratories are reporting their data at any given time,[1] whereas this analysis uses raw counts that do not correct for such variation in reporting. DEA did not provide information on which laboratories reported which records or how they varied in their reporting coverage, precluding our making similar adjustments.

Data structure & reporting

Record-level NFLIS-Drug data consist of drug reports, each representing a single instance of a specific substance detected, linked to unique seizure-level and item/sample-level identifiers. Generally, each *seizure* represents a single incident of law enforcement confiscating suspected drugs; a seizure can potentially result in multiple *items/samples* being submitted for forensic analysis, each of which in turn contains one or more substances, with each substance detected constituting a single *report*. Up to eight substances are reported for each item/sample.[1]

Potential issues with coverage & reporting in NFLIS-Drug

While the database comprehensively covers most drug chemistry analysis results, those results are themselves not representative of the overall illicit drug supply, for several reasons.

First, drugs analyzed come from law enforcement seizures. Those seizures are not a representative sampling of the illicit drug supply at consumer level. Instead, the pattern of seizures reflects a combination of a) the actual consumer-level drug supply; b) the upstream structure of the illicit drug supply chain, e.g. major smuggling routes; and c) [successful] law enforcement efforts, which may or may not be related to a) and b). Some states, such as Texas or Ohio, may thus be over-represented in the database due to more aggressive enforcement efforts combined with being along major drug smuggling routes.

Second, the seizures included range from personal possession to large-scale seizures, and the number of samples reported from each seizure may not correspond to the size of the seizure – in principle a personal possession seizure could result in multiple samples reported, while a large-scale drug bust with hundreds of kilos of drugs may yield fewer reported samples. It is thus unclear from the available data what size of seizure (or where in the illicit supply chain) any given record comes from, and how this may reflect the broader drug supply.

Third, laboratories vary in how they define items/samples.[1] For instance, some laboratories may consider separate pills or even separate bags of powder packaged together to be a single item, whereas others may only consider each pill or bag to be a single item. Analysis of co-occurrence based on shared item/sample-level identifiers thus may not strictly reflect actual physical mixtures or combinations of substances, as opposed to broader co-presence. Furthermore, reporting in NFLIS is limited to 8 substances per sample; if more than 8 substances are detected, only 8 will be reported.

Fourth, analysis is not standardized across laboratories. Testing procedures, criteria, and thresholds may vary between laboratories.[1] Laboratories also do not necessarily have the capability to test for and identify every substance present in a sample; sometimes the necessary equipment, assays, references, etc. are not available. This issue primarily affects detection of less common substances, such as novel psychoactive substances or rare fentanyl analogues; detection of major drug categories, such as those included in our analysis, is not generally an issue. Importantly, most common forms of fentanyl have been systematically screened for throughout the entire study period (2013-2023).[5] Limitations in laboratory analyses are thus unlikely to substantially affect the results presented in this article.

Fifth, even aside from laboratory capabilities, there is substantial under-reporting in NFLIS-Drug due to analysis and reporting being driven primarily by law enforcement priorities. For instance, not all drugs seized are submitted for testing, with seizures intended to be used as evidence in legal proceedings generally prioritized. In analyzing samples or reporting substances detected, laboratories may prioritize identifying or reporting substances associated with harsher criminal penalties, such as fentanyl or Schedule I controlled substances, whereas non-controlled substances may often not be tested for (or not reported even if detected). To further complicate this challenge, these law enforcement priorities vary by jurisdiction – such as due to state-level controls on or decriminalization of certain substances – and over time.

For these reasons, NFLIS-Drug reports present only a partial and approximate picture of the overall illicit drug supply. Nevertheless, in the absence of any large-scale, systematic monitoring or regulation of the drug supply, NFLIS-Drug remains an essential (and the most comprehensive) source of information for identifying trends and patterns in the drug supply over time in the United States.

Data fields available

In addition to substance detected, location (state/county) and time (year/month) of seizure, and identifiers, the NFLIS-Drug records provided by DEA also included substance form (e.g., powder, pill, liquid). However, 52.3% of the drug reports were missing this data, so we excluded it from this analysis.

NFLIS-Drug also collects information on purity and sample weight; however, these data fields suffer substantial missingness and are of limited quality, with recording and reporting protocols varying widely across laboratories.[1] For instance, some laboratories may report total quantity of substance found as sample weight (e.g. a 5 lb bag of powder), whereas others may only report the quantity actually analyzed (e.g. a 0.5g sample). Due to concerns over data quality, DEA did not provide these data fields in the drug reports analyzed here.

Crucially, NFLIS-Drug does not include information on original drug identities (e.g., 'seized as' or 'sold as').

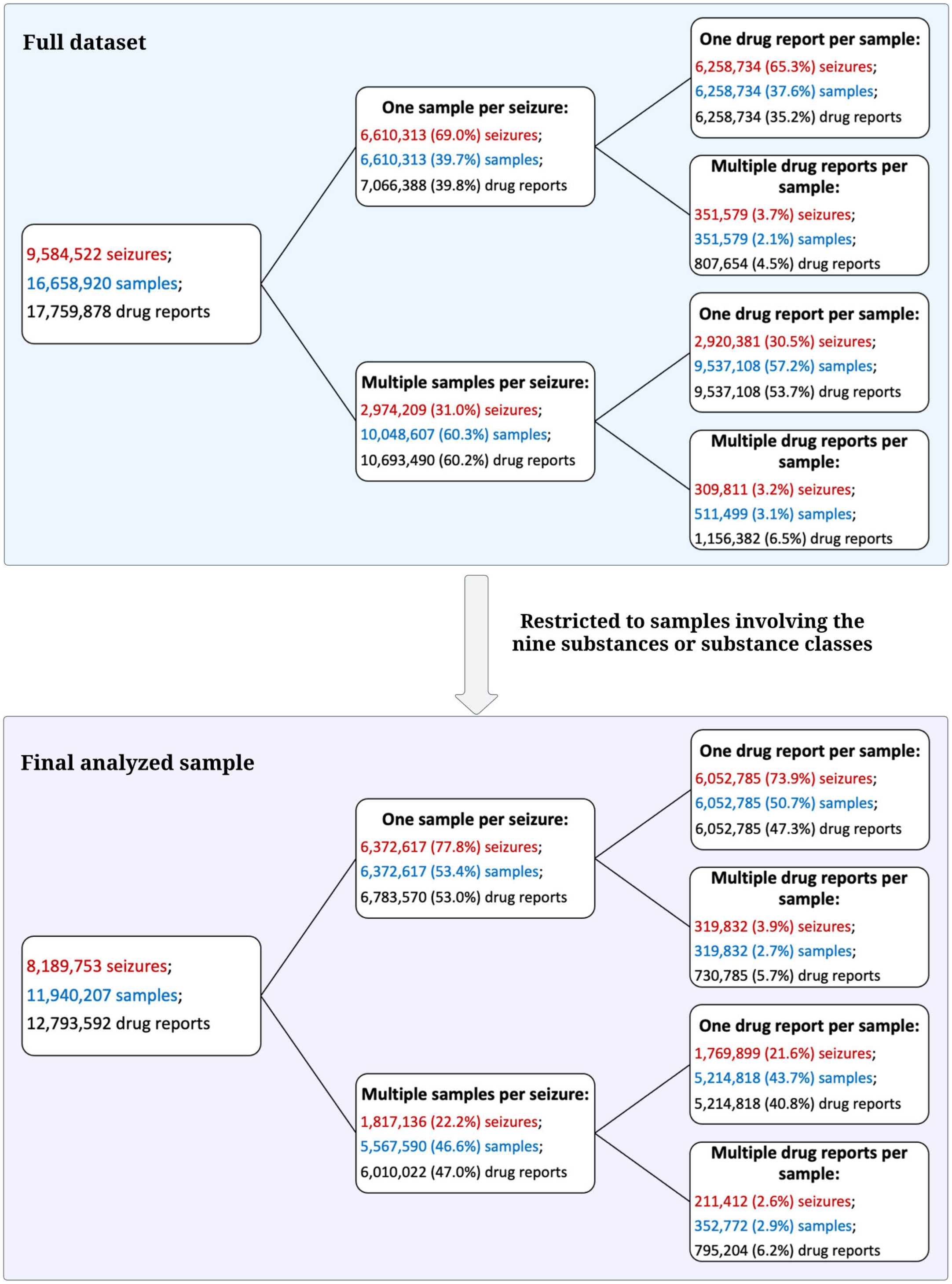

Supplementary Figure 1. Frequencies of seizures, samples, and drug reports based on the multi-level data structure among all NFLIS data from 2013-2023 across 50 states and the District of Columbia. Note that the data exclude approx. 425,000 reports from Queens County, NY, that were provided only in aggregate form.

Supplementary Table 1. Substance names reported in the NFLIS drug reports and their categorized substance/substance class.
*Note that while some substance classes are identified as 'prescription' because the substances included are available by prescription, the actual samples analyzed and reported may be illicitly manufactured.

Categorized substance / substance class	Substance name
**Heroin**	heroin
**Prescription* opioids**	oxycodone
	hydrocodone
	buprenorphine
	morphine
	methadone
	hydromorphone
	oxymorphone
**Hallucinogens**	psilocin/psilocybin
	psilocin/psilocibin
	psilocin
	psilocibin
	psilocybin
	lysergic acid diethylamide (lsd)
	Lsd
	25i-nbome
	25c-nbome
	dimethyltryptamine (dmt)
	dimethyltryptamine
	dmt
	pcp
	phencyclidine (pcp)
	3cl-pcp
**Club drugs**	ketamine
	3,4-methylenedioxyamphetamine (mda)
	ethylone
	mda
	mdma
	methylone (mdmc)
	n-cyclohexylmethylone
**Methamphetamine**	methamphetamine
	amphetamines/methamphetamines
**Cocaine**	cocaine
**Prescription* stimulants**	amphetamine
	lisdexamfetamine
	methylphenidate
**Prescription* benzodiazepines**	alprazolam
	clonazepam
	diazepam
	lorazepam
**Cannabinoids**	cannabidiol
	cannabinol
	cannabis/thc
	delta-8-tetrahydrocannabinol
	tetrahydrocannabinol/tetrahydrocannabinolic acid
**Fentanyl and fentanyl-related**	fentanyl
	Anpp
	acetyl fentanyl
	carfentanil
	fluorofentanyl
	furanyl fentanyl
	para-fluorofentanyl
	valeryl fentanyl
	fuoroisobutyryl fentanyl
	4-fluoroisobutyryl fentanyl
	cyclopropyl fentanyl
	acryl fentanyl
	methoxyacetyl fentanyl

	3-methylfentanyl
	phenethyl 4-anpp
	butyryl fentanyl
	benzylfentanyl
	fluorobutyryl fentanyl
	phenyl fentanyl
	fluoro 4-anpp
	p-fluorobutyryl fentanyl
	cyclopropyl/crotonyl fentanyl
	para-fluoro furanyl fentanyl
	fluoro furanyl fentanyl
	fentanyl-related compound (unspecified)
	furanyl benzyl fentanyl
	para-fluoro 4-anpp
	crotonyl fentanyl
	n-methyl norfentanyl
	chlorofentanyl
	fluorophenethyl 4-anpp
	ortho-chlorofentanyl
	ortho-fluorofentanyl
	p-fluoro benzyl fentanyl
	para-chlorofentanyl
	norfentanyl
	isobutyryl fentanyl
	cis-3-methylfentanyl
	thiofuranyl fentanyl
	trans-3-methylfentanyl
	1-phenethyl-4-piperidone
	trahydrofuran fentanyl
	fentanyl carbamate
	furanyl/3-furanyl fentanyl
	isovaleryl fentanyl
	n-(1-phenethyl-4-piperidyl)-n-phenethylpropanamide
	beta-hydroxythiofentanyl
	p-fluoro cyclopropyl benzyl fentanyl
	meta-fluorofentanyl
	meta-fluoro 4-anpp
	methyl acetyl fentanyl
	n-phenyl-4-piperidinamine
	1-phenethyl-4-propionyloxypiperidine
	acetyl-alpha-methylfentanyl
	chloro 4-anpp
	para-fluoro valeryl fentanyl
	alpha'-methyl butyryl fentanyl
	1-propionyl-4-anilinopiperidine
	3-furanyl fentanyl
	4-carbomethoxy 4-anpp
	4-fluorophenethyl 4-anpp
	beta-methyl acetyl fentanyl
	flurobutyryl/fluoroisobutyryl fentanyl
	ortho-chloro 4-anpp
	para-methylfentanyl
	3',4'-dimethoxy fentanyl
	methylfentanyl
	ortho-methyl 4-anpp
	n-propionyl norfentanyl
	1-phenethyl-4-hydroxypiperidine
	4'-methyl acetyl fentanyl
	beta-methylfentanyl
	cyclopentyl fentanyl
	methyl 4-anpp
	methyl cyclopropyl fentanyl
	n-benzyl fluoro norfentanyl
	n,n-phenethyl-phenyl-1-(2-phenylethyl)piperidin-4-amine
	o-fluoro acrylfentanyl
	ortho-fluoro furanyl fentanyl

	1-boc-4-piperidone
	3-fluorofentanyl
	3-fluoroisobutyryl fentanyl
	benzodioxole fentanyl
	ortho-fluoro 4-anpp
	ortho-methyl acetyl fentanyl
	sufentanil
	3-phenylpropanoyl fentanyl
	acetyl norfentanyl
	alpha-methylfentanyl
	benzoylbenzyl fentanyl
	benzyl acryl fentanyl
	fluoro 4-anilinopiperidine
	meta-fluoro furanyl fentanyl
	meta-methyl 4-anpp
	methoxybutyryl fentanyl
	n-boc norfentanyl
	ocfentanil
	p-methoxybutyryl fentanyl
	para-methoxy furanyl fentanyl
	tetrahydrothiofuranyl fentanyl
	valeryl/isovaleryl fentanyl
	4-anpp
	1-boc-4-(4-fluoroanilino)piperidine
	2-chloro 4-anpp
	2-fluoro 4-anpp
	3-methylthiofentanyl
	acryl-alpha-methylfentanyl
	alfentanil
	benzylacryl fentanyl
	bipiperidinyl 4-anpp
	ethyl 4-anpp
	fluoro acetyl fentanyl
	fluoro phenethyl 4-anpp
	fluoro valeryl fentanyl
	lofentanil
	n-(1-phenethyl-4-piperidyl)-n-phenethylpropionamide
	n-(1-tert-butyl-4-piperidyl)-n-phenylpropanamide
	n-benzyl furanyl fentanyl
	n-ethyl norfentanyl
	remifentanil
	tetrahydrofuranyl fentanyl
	thenylfentanyl
	thiofentanyl
	alpha'-methylbutyryl fentanyl
	alpha-methylthiofentanyl
	beta'-phenyl fentanyl
	beta-hydroxy-3-methylfentanyl
	beta-hydroxyfentanyl
	meta-fluoroisobutyryl fentanyl
	ortho-fluoroacryl fentanyl
	ortho-fluorobutyryl fentanyl
	ortho-fluoroisobutyryl fentanyl
	ortho-methoxy furanyl fentanyl
	ortho-methoxybutyryl fentanyl
	para-fluoro benzyl fentanyl
	para-fluoro cyclopropyl benzyl fentanyl
	para-fluoroacryl fentanyl
	para-fluorobutyryl fentanyl
	para-fluoroisobutyryl fentanyl
	para-methoxybutyryl fentanyl
	para-fluoro phenethyl 4-anpp

Supplementary Table 2. Total number of drug reports and samples for each substance and the number of samples with co-occurring fentanyl in the United States, 2013-2023

Substance	Number of drug reports	Number of samples	Number of samples with fentanyl co-occurrence
**Fentanyl**	1125645	1010939	---
**Methamphetamine**	3850926	3850672	18628
**Cannabinoids**	3203792	3177205	1580
**Cocaine**	2014562	2012480	27621
**Heroin**	1326703	1325203	179284
**Prescription opioids**	797185	795166	2909
**Prescription benzodiazepines**	483483	482977	785
**Prescription stimulants**	144830	142107	108
**Hallucinogens**	150382	144235	131
**Club drugs**	99792	97025	2121

Supplementary Table 3. STROBE checklist

Item	No	Recommendation	Page No
**Title and abstract**	1	(*a*) Indicate the study's design with a commonly used term in the title or the abstract	Page 1
		(*b*) Provide in the abstract an informative and balanced summary of what was done and what was found	Page 1
**Introduction**			
Background/rationale	2	Explain the scientific background and rationale for the investigation being reported	Intro, para 1-4
Objectives	3	State specific objectives, including any prespecified hypotheses	Intro, para 5
**Methods**			
Study design	4	Present key elements of study design early in the paper	Methods, para 1-5
Setting	5	Describe the setting, locations, and relevant dates, including periods of recruitment, exposure, follow-up, and data collection	Methods, para 1-3; Supplementary Methods
Participants	6	(*a*) Give the eligibility criteria, and the sources and methods of selection of participants	Methods, para 1-3; Supplementary Methods
Variables	7	Clearly define all outcomes, exposures, predictors, potential confounders, and effect modifiers. Give diagnostic criteria, if applicable	Methods, para 1-3; Supplementary Methods
Data sources/ measurement	8	For each variable of interest, give sources of data and details of methods of assessment (measurement). Describe comparability of assessment methods if there is more than one group	Methods, para 1-3; Supplementary Methods
Bias	9	Describe any efforts to address potential sources of bias	Methods, para 5; Limitations
Study size	10	Explain how the study size was arrived at	Methods, para 3-4; Supplementary Figure 1
Quantitative variables	11	Explain how quantitative variables were handled in the analyses. If applicable, describe which groupings were chosen and why	Methods, para 3-4; Supplementary Methods
Statistical methods	12	(*a*) Describe all statistical methods, including those used to control for confounding	Methods, para 3-5;
		(*b*) Describe any methods used to examine subgroups and interactions	Methods, para 3-5;
		(*c*) Explain how missing data were addressed	Methods, para 1-5;
		(*d*) If applicable, describe analytical methods taking account of sampling strategy	Methods, para 3-5;
		(*e*) Describe any sensitivity analyses	Methods, para 3-5;
**Results**			
Participants	13	(a) Report numbers of individuals at each stage of study—eg numbers potentially eligible, examined for eligibility, confirmed eligible, included in the study, completing follow-up, and analysed	Results para 1-2 Supplementary Figure 1
		(b) Give reasons for non-participation at each stage	Results para 1-2
		(c) Consider use of a flow diagram	Supplementary Figure 1
Descriptive data	14	(a) Give characteristics of study participants (eg demographic, clinical, social) and information on exposures and potential confounders	Results para 2
		(b) Indicate number of participants with missing data for each variable of interest	Supplementary Methods
Outcome data	15	Report numbers of outcome events or summary measures	Results para 2
Main results	16	(*a*) Give unadjusted estimates and, if applicable, confounder-adjusted estimates and their precision (eg, 95% confidence interval). Make clear which confounders were adjusted for and why they were included	Results para 3-7
		(*b*) Report category boundaries when continuous variables were categorized	Not relevant
		(*c*) If relevant, consider translating estimates of relative risk into absolute risk for a meaningful time period	Not relevant
Other analyses	17	Report other analyses done—eg analyses of subgroups and interactions, and sensitivity analyses	S3. Additional Results
Discussion			
**Key results**	18	Summarise key results with reference to study objectives	
Limitations	19	Discuss limitations of the study, taking into account sources of potential bias or imprecision. Discuss both direction and magnitude of any potential bias	Discussion, para 9-14
Interpretation	20	Give a cautious overall interpretation of results considering objectives, limitations, multiplicity of analyses, results from similar studies, and other relevant evidence	Discussion, para 1-8
Generalisability	21	Discuss the generalisability (external validity) of the study results	Discussion, para 7
**Other information**			
Funding	22	Give the source of funding and the role of the funders for the present study and, if applicable, for the original study on which the present article is based	Acknowledgements

Supplementary Table 4. Mann-Kendall trend test results for monthly proportion of each substance category with co-occurring fentanyl, aggregated nationally, 2013-2023

Substance category	Tau	p value
Cannabinoids	0.78713	<0.0001
Club drugs	0.49364	<0.0001
Cocaine	0.88252	<0.0001
Hallucinogens	0.53836	<0.0001
Heroin	0.94569	<0.0001
Methamphetamine	0.89029	<0.0001
Prescription benzodiazepines	0.6464	<0.0001
Prescription opioids	0.76338	<0.0001
Prescription stimulants	0.36302	<0.0001

Supplementary Table 5. Mann-Kendall trend test results for yearly proportion of each substance category with co-occurring fentanyl, by state and substance category, 2013-2023. Tabulated values are *p* values for the Mann-Kendall test. Color indicates co-occurrence trend direction while intensity indicates significance – light blue indicates decreasing trend with $p \geq 0.05$ and orange indicates increasing trend with different levels of statistical significance: $p \geq 0.05$; $0.05 > p \geq 0.001$; $0.001 > p \geq 0.0001$; $p < 0.0001$. NaN values indicate insufficient data to calculate the Mann-Kendall test statistic.

	1	2	3	4	5	6	7	8	9
**AK**	NaN	0.16	0.06	NaN	0.016	0.005	NaN	0.64	NaN
**AL**	0.023	0.11	0.0008	NaN	<0.0001	0.003	0.64	0.24	NaN
**AR**	NaN	0.87	0.008	NaN	0.009	0.003	NaN	NaN	0.43
**AZ**	0.014	0.52	0.0002	0.15	0.0007	<0.0001	0.032	0.23	0.15
**CA**	0.003	0.10	0.0002	0.06	<0.0001	<0.0001	0.001	0.0001	0.08
**CO**	0.10	0.24	0.008	NaN	0.0002	0.0002	0.035	0.009	0.64
**CT**	0.032	0.11	0.001	0.16	<0.0001	0.55	0.27	0.94	NaN
**DC**	0.24	NaN	0.0004	NaN	<0.0001	0.67	NaN	NaN	NaN
**DE**	NaN	0.24	0.15	NaN	0.0008	0.64	NaN	NaN	NaN
**FL**	0.01	0.14	0.002	0.43	<0.0001	0.0002	0.13	0.013	0.35
**GA**	0.87	0.87	0.015	NaN	0.0006	0.01	0.35	0.10	NaN
**HI**	NaN	NaN	0.009	NaN	0.11	0.028	NaN	NaN	NaN
**IA**	0.87	0.32	0.16	0.43	0.006	0.08	NaN	0.93	NaN
**ID**	0.06	0.035	0.028	NaN	0.001	0.007	0.08	0.009	NaN
**IL**	0.006	0.004	<0.0001	0.15	<0.0001	0.004	0.07	0.001	0.64
**IN**	0.028	0.87	0.004	NaN	0.0002	0.004	0.87	0.32	NaN
**KS**	0.005	0.10	0.001	0.27	0.0008	0.0004	0.06	0.004	0.35
**KY**	0.001	0.024	0.0008	0.10	<0.0001	0.0002	0.022	0.0002	0.07
**LA**	0.27	0.16	0.003	NaN	0.0004	0.045	NaN	0.008	NaN
**MA**	0.07	0.009	0.64	0.43	0.0006	0.53	0.17	0.81	NaN
**MD**	0.001	0.31	0.0006	0.07	<0.0001	0.002	0.015	0.07	0.15
**ME**	NaN	0.87	0.004	NaN	0.0006	0.35	0.48	NaN	0.64
**MI**	0.07	0.43	0.001	NaN	<0.0001	0.005	0.32	0.27	NaN
**MN**	0.80	0.67	0.002	0.11	0.0001	0.015	0.17	0.34	NaN
**MO**	0.07	0.15	0.0002	0.87	0.002	0.001	0.004	0.0006	0.07
**MS**	0.028	0.64	0.0002	0.64	0.0004	0.0001	0.27	0.08	NaN
**MT**	0.27	NaN	0.27	NaN	0.001	0.001	NaN	0.64	NaN
**NC**	0.23	0.52	0.14	NaN	0.06	0.94	0.87	0.51	NaN
**ND**	0.64	0.64	0.84	NaN	0.41	0.41	0.87	0.87	NaN
**NE**	0.64	NaN	0.045	NaN	0.005	0.021	0.64	0.84	NaN
**NH**	0.62	0.62	0.64	0.64	0.35	1.00	1.00	0.87	0.32
**NJ**	0.004	0.10	<0.0001	0.002	<0.0001	0.14	0.0007	0.001	0.021
**NM**	0.06	0.045	0.002	0.43	0.0002	0.001	0.24	0.35	NaN
**NV**	0.10	0.87	0.0008	NaN	0.0007	0.002	NaN	0.036	NaN
**NY**	0.87	0.31	0.002	0.81	<0.0001	0.015	0.27	0.001	0.24
**OH**	0.001	0.006	<0.0001	0.036	0.0002	0.0006	<0.0001	0.003	0.13
**OK**	0.014	0.15	0.013	0.27	0.0001	0.002	0.032	0.07	NaN
**OR**	0.64	0.35	0.009	NaN	<0.0001	0.0002	0.55	0.67	NaN
**PA**	0.0002	0.006	0.0003	0.049	<0.0001	<0.0001	0.07	0.16	0.049
**RI**	1.00	0.60	0.0004	NaN	0.06	0.32	NaN	NaN	NaN
**SC**	0.016	0.26	0.0002	0.64	0.0003	0.0001	0.003	0.20	0.81
**SD**	NaN	NaN	NaN	NaN	0.005	0.10	NaN	NaN	NaN
**TN**	0.001	0.07	0.006	0.27	0.002	0.015	0.84	0.52	NaN
**TX**	0.016	0.021	0.0002	0.15	<0.0001	0.0004	0.51	0.008	NaN
**UT**	0.15	0.035	0.017	0.15	0.005	0.008	0.009	0.06	0.06
**VA**	0.002	0.005	0.0002	0.81	<0.0001	0.0002	0.02	0.0002	0.32
**VT**	NaN	0.74	0.53	NaN	0.043	0.48	NaN	1.00	NaN
**WA**	1.00	0.84	0.004	NaN	0.001	0.038	NaN	0.27	NaN
**WI**	0.19	0.0003	0.001	NaN	<0.0001	0.0004	NaN	0.07	0.10
**WV**	0.64	0.42	0.015	0.87	0.44	0.18	0.55	0.74	NaN
**WY**	NaN	NaN	NaN	NaN	NaN	NaN	NaN	NaN	NaN

Substance Key: 1. Cannabinoids, 2. Club drugs, 3. Cocaine, 4. Hallucinogens, 5. Heroin, 6. Methamphetamine, 7. Prescription benzodiazepines, 8. Prescription opioids, 9. Prescription stimulants

(a)

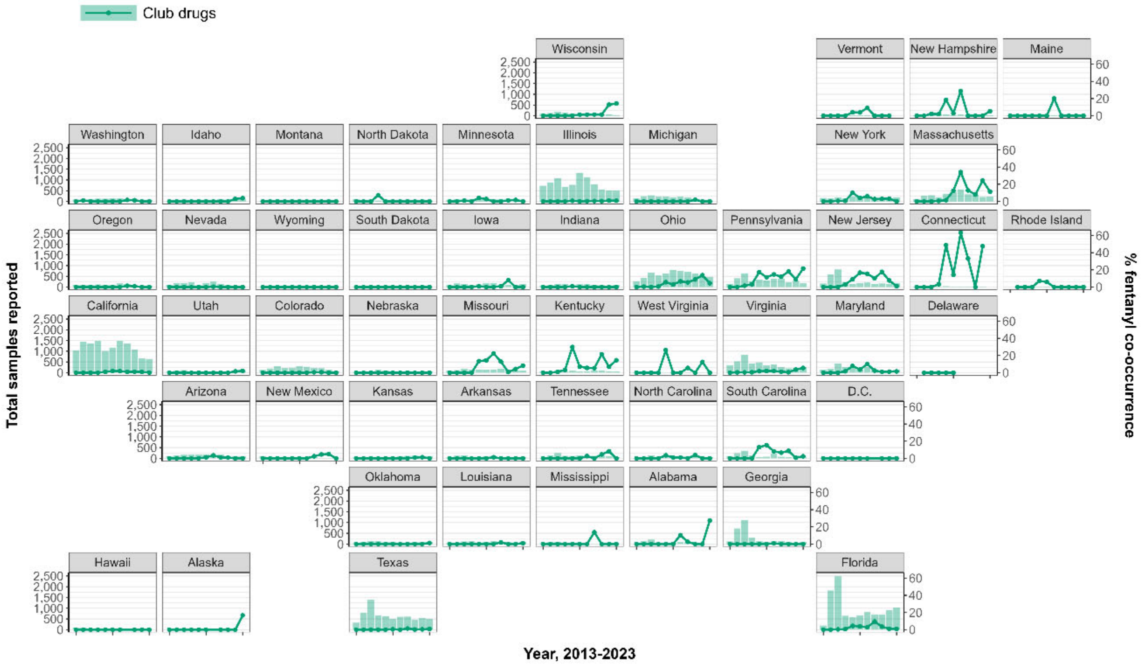
Club drugs
Wisconsin
Vermont
New Hampshire
Maine
Washington
Idaho
Montana
North Dakota
Minnesota
Illinois
Michigan
New York
Massachusetts
Oregon
Nevada
Wyoming
South Dakota
Iowa
Indiana
Ohio
Pennsylvania
New Jersey
Connecticut
Rhode Island
California
Utah
Colorado
Nebraska
Missouri
Kentucky
West Virginia
Virginia
Maryland
Delaware
Arizona
New Mexico
Kansas
Arkansas
Tennessee
North Carolina
South Carolina
D.C.
Oklahoma
Louisiana
Mississippi
Alabama
Georgia
Hawaii
Alaska
Texas
Florida
0
500
1,000
1,500
2,000
2,500
0
20
40
60
Total samples reported
% fentanyl co-occurrence
Year, 2013-2023

(b)

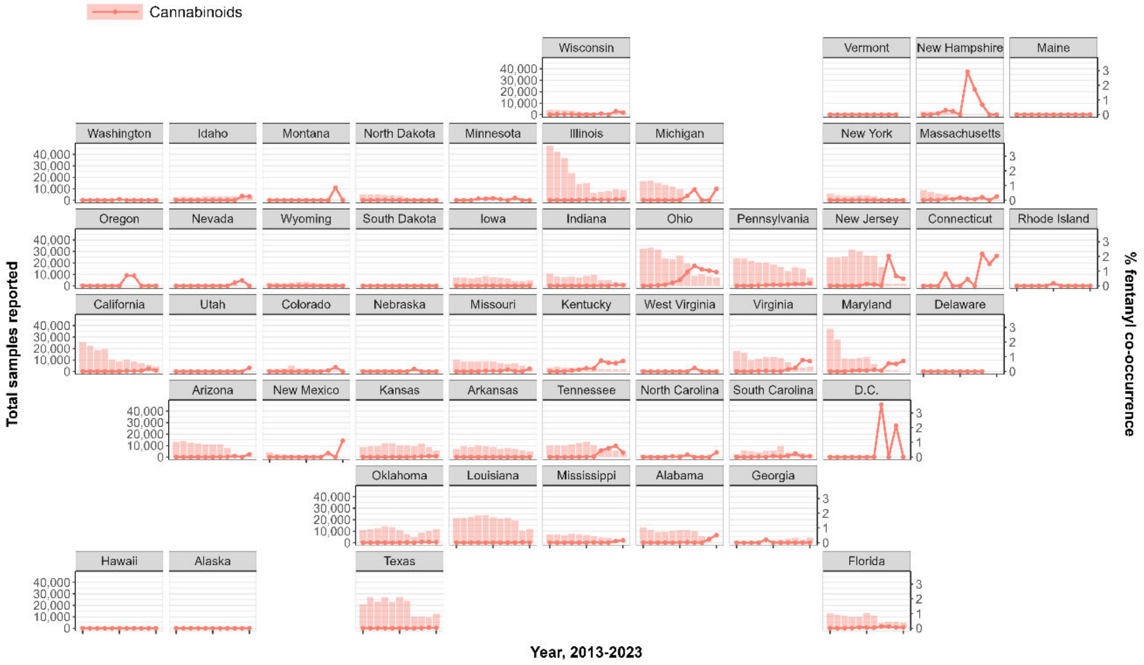
Cannabinoids
Wisconsin
Vermont
New Hampshire
Maine
Washington
Idaho
Montana
North Dakota
Minnesota
Illinois
Michigan
New York
Massachusetts
Oregon
Nevada
Wyoming
South Dakota
Iowa
Indiana
Ohio
Pennsylvania
New Jersey
Connecticut
Rhode Island
California
Utah
Colorado
Nebraska
Missouri
Kentucky
West Virginia
Virginia
Maryland
Delaware
Arizona
New Mexico
Kansas
Arkansas
Tennessee
North Carolina
South Carolina
D.C.
Oklahoma
Louisiana
Mississippi
Alabama
Georgia
Hawaii
Alaska
Texas
Florida
0
10,000
20,000
30,000
40,000
0
1
2
3
Total samples reported
% fentanyl co-occurrence
Year, 2013-2023

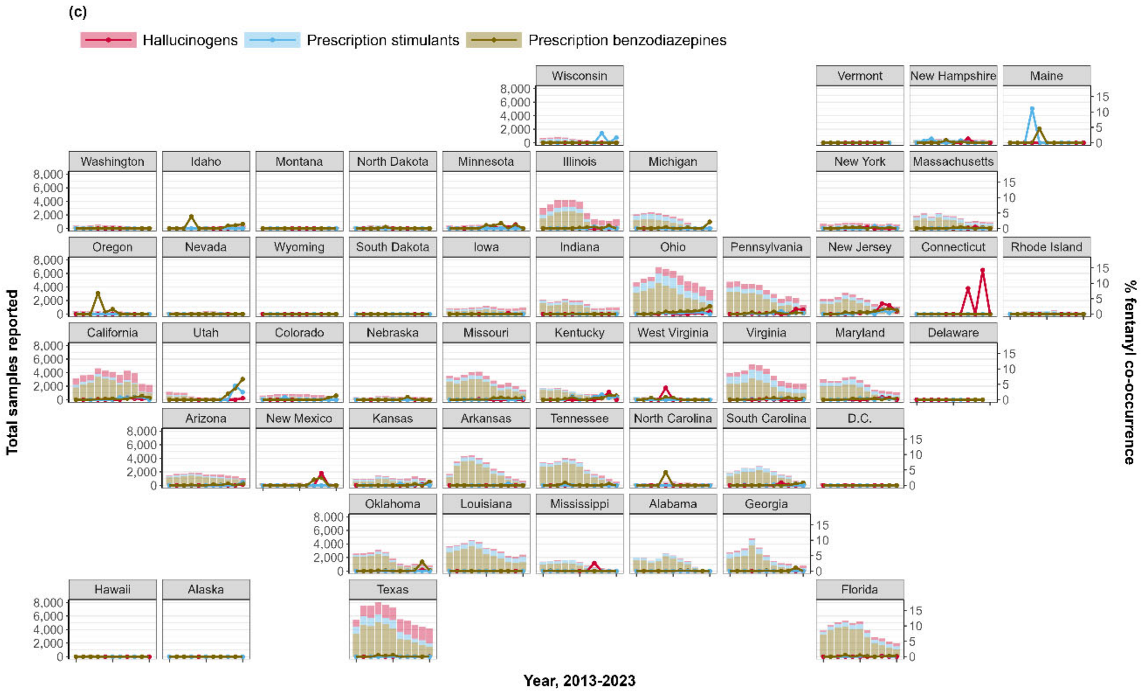

Supplementary Figure 2. State-level trends in the yearly proportion of different substance samples with co-occurring fentanyl in the United States, 2013-2023. The following data points were excluded from the figure since the number of total tested samples was ≤5, which caused unstable estimates. For club drugs: DE (year: 2019) and CT (2023). For prescription benzodiazepines: ME (2022). Fentanyl co-occurrence among prescription benzodiazepines samples was 25.0% for CT in the year 2022.

References

1. Pitts WJ, Heller D, Smiley-McDonald H, et al. Understanding research methods, limitations, and applications of drug data collected by the National Forensic Laboratory Information System (NFLIS-Drug). *J Forensic Sci*. 2023;68(4):1335-1342. doi:10.1111/1556-4029.15269
2. Diversion Control Division. National Forensic Laboratory Information System: NFLIS-Drug 2022 Midyear Report. U.S. Department of Justice, U.S. Drug Enforcement Administration. April 2023. Accessed June 26, 2023. https://www.nflis.deadiversion.usdoj.gov/publicationsRedesign.xhtml
3. U.S. Drug Enforcement Administration. *National Forensic Laboratory Information System: Year 2013 Annual Report*.; 2014.
4. Diversion Control Division. *National Forensic Laboratory Information System: NFLIS-Drug 2022 Annual Report*.; 2023.
5. Drug Enforcement Administration Diversion Control Division. *NFLIS Brief: Fentanyl, 2001-2015*.; 2017. https://www.deadiversion.usdoj.gov/nflis/2017fentanyl.pdf
